# Homotypic cell competition regulates proliferation and tiling of zebrafish pigment cells during colour pattern formation

**DOI:** 10.1038/ncomms11462

**Published:** 2016-04-27

**Authors:** Brigitte Walderich, Ajeet Pratap Singh, Prateek Mahalwar, Christiane Nüsslein-Volhard

**Affiliations:** 1 Max Planck Institute for Developmental Biology, Spemannstrasse 35, 72076 Tübingen, Germany

## Abstract

The adult striped pattern of zebrafish is composed of melanophores, iridophores and xanthophores arranged in superimposed layers in the skin. Previous studies have revealed that the assembly of pigment cells into stripes involves heterotypic interactions between all three chromatophore types. Here we investigate the role of homotypic interactions between cells of the same chromatophore type. Introduction of labelled progenitors into mutants lacking the corresponding cell type allowed us to define the impact of competitive interactions via long-term *in vivo* imaging. In the absence of endogenous cells, transplanted iridophores and xanthophores show an increased rate of proliferation and spread as a coherent net into vacant space. By contrast, melanophores have a limited capacity to spread in the skin even in the absence of competing endogenous cells. Our study reveals a key role for homotypic competitive interactions in determining number, direction of migration and individual spacing of cells within chromatophore populations.

## Abstract (web summary)

Melanophores, iridophores and xanthophores are pigment-cell types that interact to form the stripes in zebrafish. Here, the authors study the interaction between cells of the same kind and show that each pigment-cell type covers the skin by contact based competition.

## Graphical abstract

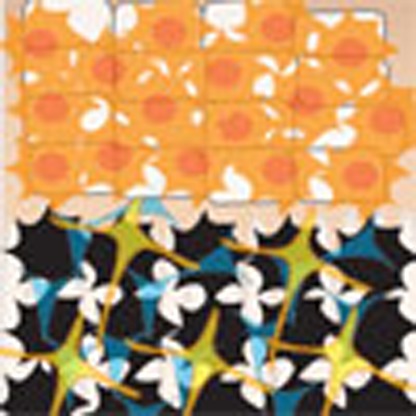

Colour patterns are widespread in the animal kingdom and not only protect against harmful radiation, but also serve as recognition signals in intra- and interspecies communication. The zebrafish, *Danio rerio*, has emerged as the vertebrate model organism to study the mechanisms underlying colour pattern formation^1^,^2^,^3^,^4^. The characteristic pattern of alternating horizontal dark and light stripes represents a system of three different pigment-cell types distributed in superimposed layers in the skin. In the dark stripes, black melanophores are covered by a middle layer of blue iridophores and a thin top layer of yellow xanthophores. In the light stripes, a compact layer of xanthophores covers silvery iridophores.

The adult pigmentation pattern is formed during metamorphosis, a period between ∼3 and 6 weeks of development. Iridophores and melanophores are derived from stem cells located at the segmentally reiterated dorsal root ganglia (DRG) of the peripheral nervous system^5^,^6^,^7^. Iridophores emerge in the skin along the horizontal myoseptum; they proliferate and spread as densely connected cells forming the first light stripe. They spread further, dorsally and ventrally, as a loose net of cells into the regions where the dark stripes will form and then again undergo patterned aggregation to form new light stripes^5^. Melanoblasts migrate and proliferate along the peripheral neurons innervating the skin and emerge *in situ* in the skin where they differentiate and expand to fill in the dark stripes^5^,^6^,^7^. Most adult xanthophores arise from larval xanthophores, which begin to divide at the onset of metamorphosis and cover the entire body of the fish^8^,^9^.

While each pigment-cell type is distributed in a single cell wide layer, xanthophores and iridophores display different morphologies depending on their position in the pattern: in the dark stripes stellate xanthophores form a net-like structure and loose iridophores appear blue, whereas densely packed, silvery iridophores are tightly associated with compact xanthophores in the light stripes^8^,^10^,^11^,^12^. The establishment of organized cell morphologies indicates close cell–cell communication between skin layers, and is essential for the sharpness and brightness of the striped pattern.

Mutants lacking one or more of the pigment-cell types are not able to produce the striped pattern correctly (for example, *nacre* (encoding Mitfa) mutants that lack melanophores, *pfeffer*/*fms* (encoding Csf1rA) mutants that lack xanthophores, and *shady* (encoding Ltk), *rose* (encoding Ednrb1Ba) and *transparent* (encoding Mpv17) mutants where iridophores are absent or strongly reduced)^13^,^14^,^15^,^16^,^17^. In all these cases the two remaining chromatophore types form an irregular, residual striped pattern. Supplementing the missing cell type in chimeric animals obtained by blastula transplantations can locally restore a normal pattern^12^,^17^,^18^. This indicates that heterotypic interactions between the three cell types are required to form a normal pattern. Analyses of mutants lacking one of the pigment-cell types, as well as ablation experiments, have suggested the presence of several attractive and repulsive signals between chromatophores, which act over long or short ranges during stripe formation^12^,^19^,^20^. In the absence of xanthophores, melanophore numbers are reduced, stripes break up into spots, and ectopic melanophores remain scattered in the light stripe region. In iridophore mutants, the number of melanophores is also strongly reduced, and only the first two dark stripes form broken into spots^5^,^12^. In the absence of two pigment-cell types, remaining iridophores (in *nacre*; *pfeffer* mutants) and xanthophores (in *shady*; *pfeffer*) cover the flank of the fish, whereas the melanophores in the absence of iridophores and xanthophores (*shady*; *pfeffer*) are reduced in number and scattered in lower than normal density^12^. These observations indicate a strong dependence of melanophore survival on interactions with the other cell types, whereas iridophores and xanthophores display a more autonomous behaviour.

Previous analyses of chimeras generated between wild type and mutants lacking one of the three cell types placed emphasis on the interactions among different pigment cells (heterotypic interactions)^12^,^18^. The role of homotypic interactions (between cells of the same type) remains poorly understood. *In vitro* observations of interactions between isolated pigment cells did not uncover any obvious response between cells of the same type, although an interaction response between melanophores and xanthophores has been detected^21^. Genetic analyses also have suggested that homotypic interactions exist among melanophores and xanthophores^18^,^22^. Here, we analyse the cell-level outcome of the homotypic interactions among the chromatophores *in vivo* through the generation of chimeric animals, and we corroborate our findings with results from regeneration experiments. We investigate the proliferation and spread of labelled chromatophore clusters in the presence or absence of endogenous cells within a layer. For all pigment-cell types, we observe an increase in the average size of clusters in environments lacking the respective cell type. This indicates that there is competition between pigment cells during normal development. Xanthophores and iridophores have an intrinsic tendency to proliferate and evenly fill the space in the skin, whereas melanophores have only a restricted potential to proliferate and spread in the skin. In addition, our observation that clusters of all three pigment-cell types filled the chromatophore-devoid region in their neighbourhood as coherent nets with normal density (rather than dispersing uniformly into all available vacant space), suggests that there are contact dependent homotypic interactions between chromatophores. In all cases, donor-derived chromatophores locally restored the organization of the host chromatophores, which resulted in a normal pattern. We conclude that, whereas changes in pigment-cell morphologies during stripe formation are regulated by heterotypic interactions^22^,^23^, pigment-cell proliferation and dispersal in the skin are predominantly regulated by homotypic competition.

## Results

### Blastomere transplantations

We transplanted a small number of blastomeres to obtain single-labelled pigment-cell progenitors in wild type or mutant embryos. Using appropriate markers, we selected chimeric individuals for the presence of small clusters of donor-derived pigment cells at larval stages (5 days post fertilization (dpf)) or at the onset of metamorphosis (21 dpf) and traced their progeny to adulthood. Blastomere transplantations between wild-type embryos resulted in clusters of pigment cells that resemble Cre-induced clones^5^ in shape and size, indicating that frequently a single progenitor cell had given rise to the clusters, and that in many cases the clusters were clonal. In experiments that allowed us to follow more than one type of pigment cell it became apparent that in most cases the donor cells gave rise to multipotent neural crest progenitors of all three cell types.

### Homotypic interactions among xanthophores

**Figure 1:**
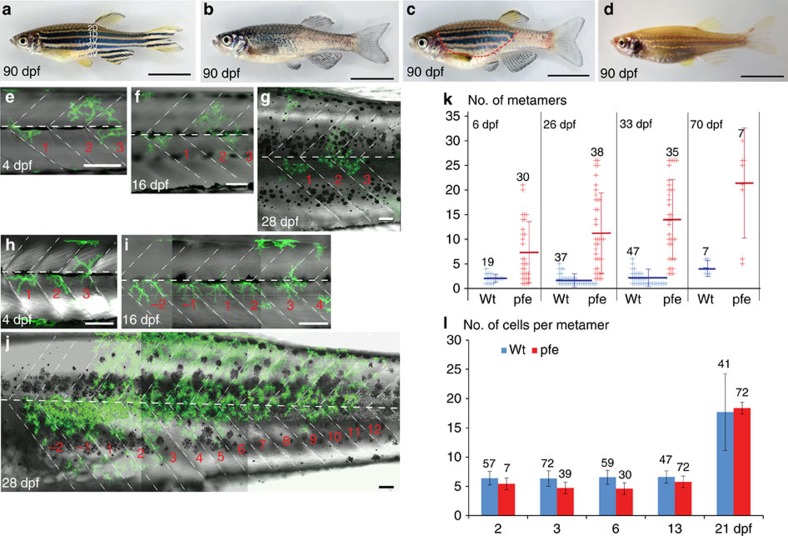
Development of xanthophore clusters in wild type and *pfeffer.* (**a**–**d**) Stripe pattern in 3-month-old (**a**) wild-type zebrafish, (**b**) *pfeffer* mutant, (**c**) chimera obtained by blastomere transplantation of wild type (*Tg*(*pax7:GFP*)) into *pfeffer*; the area with donor xanthophores is demarcated by a red dashed line and (**d**) control (*albino*). In (**a**) the dark stripes nomenclature is depicted along the dorsoventral axis. Developmental profile of *Tg*(*pax7:GFP*)-labelled wild-type xanthophores (green) in a (**e**–**g**) control and (**h**–**j**) *pfeffer* chimera. Dashed white lines—vertical and horizontal myosepta. Scale bars, **a**–**d**=1 cm; **e**–**j**=100 μm. (**k**) Quantification of the number of metameres spanned by xanthophore clusters at larval stage (6 dpf) (wild type, *n*=19 clusters, 12 fishes; *pfeffer, n*=30 clusters, 18 fishes; *P*≤0.0001); 26 dpf (wild type, *n*=37 clusters, 10 fishes; *pfeffer*, *n*=38 clusters, 17 fish; *P*≤0.0001); 33 dpf (wild type, *n*=47 clusters, 10 fishes; *pfeffer*, *n*=35 clusters, 17 fishes; *P*<0.0001); 70 dpf (wild type, *n*=7 clusters, 4 fishes; *pfeffer*, *n*=7 clusters, 7 fishes; *P*=0.0077). The horizontal lines in dark blue (wild type) and red (*pfeffer*) indicate the mean value and the error bars represent standard deviation. We show that the differences between *albino* and *pfeffer* are significant at all time points investigated by using Student's *t*-test (Welsh corrected). (**l**) Number of xanthophores per metamere in wild type (blue) and *pfeffer* transplants (red). Numbers of metameres analysed are depicted in the graph, error bars represent standard deviation. No significant differences between wild-type fish (blue) and *pfeffer* transplants (red) according to Student;s *t*-test (Welsh corrected) were found for 2 dpf: *P*=0.1795 and 21 dpf: *P*=0.6123. Overall 3 dpf (*P*=<0.0001), 6 dpf (*P*=<0,0001) and 13 dpf (*P*=0.0042) were shown to be significantly different.

The dynamics of xanthophore morphogenesis during stripe formation has been described previously^8^. To specifically track the xanthophores in normal development, chimeric animals were created by transplantation of cells from transgenic *Tg*(*pax7:GFP*)^8^,^24^ blastula stage embryos into wild-type or *albino* hosts (Fig. 1a,d). In *albino* (Fig. 1d) melanophores are unpigmented^25^, allowing for better visibility of donor-derived labelled xanthophores and pigmented melanophores. We followed the pattern development of individual fish with small clusters of 1–5 xanthophores in larvae (<5 dpf) from the onset of metamorphosis until 70 dpf (*n*=47 xanthophore clusters in 12 fishes) (Fig. 1e–g; Supplementary Fig. 1). Numbers of donor-derived xanthophores in the clusters remained constant during larval stages^8^. At the onset of metamorphosis between 16 and 20 dpf, the xanthophores began to divide as did the host xanthophores^8^. Subsequently the clusters spanned over 1–4 adjacent metameres (Fig. 1k) and across several light and dark stripes along the dorsoventral axis (Supplementary Fig. 1). The rate of division was variable between different clusters, averaging about one cell division per 10 days, as was previously observed in non-chimeric fish^8^. Labelled xanthophore clusters consisted of more or less loosely connected cells.

*pfeffer* mutants lack xanthophores in adults (Fig. 1b)^18^. The introduction of xanthophore progenitors into *pfeffer* hosts can lead to large patches of stripe rescue in chimeric animals indicating that xanthophores have the ability to divide rapidly and to spread into vacant space^18^ (Fig. 1c). We analysed xanthophore clusters in chimeras obtained by transplantation of *Tg*(*pax7:GFP*) blastomeres into *pfeffer* mutant host embryos. The donor-derived xanthophores, in contrast to xanthophore clusters in wild-type hosts (Fig. 1h–k), already divided during larval stages and occupied larger areas in the skin during metamorphosis. The numbers of segments occupied by xanthophores in *pfeffer* hosts continually increased starting in larval stages (Fig. 1k). The analysis of *pfeffer* chimeras (*n*=38 clusters of xanthophores in 18 fishes) revealed that the clusters spread on average over 6 metameres during larval stages (by 6 dpf) and by the age of 70 dpf they had spread over 21 metameres (Fig. 1k). Comparing the number of xanthophores per metamere in wild-type fish and *pfeffer* chimeras (Fig. 1l) revealed that the donor-derived xanthophores eventually fully restored the xanthophore number. This indicates that in normal development competition between xanthophores limit their rate of proliferation and spreading. Recently, it has been suggested that xanthophore proliferation and differentiation is triggered by thyroid hormone stimulation at the onset of metamorphosis^9^. Our data indicate that the reduction in xanthophore cell density is the critical cue that alone can trigger xanthophore proliferation.

**Figure 2:**
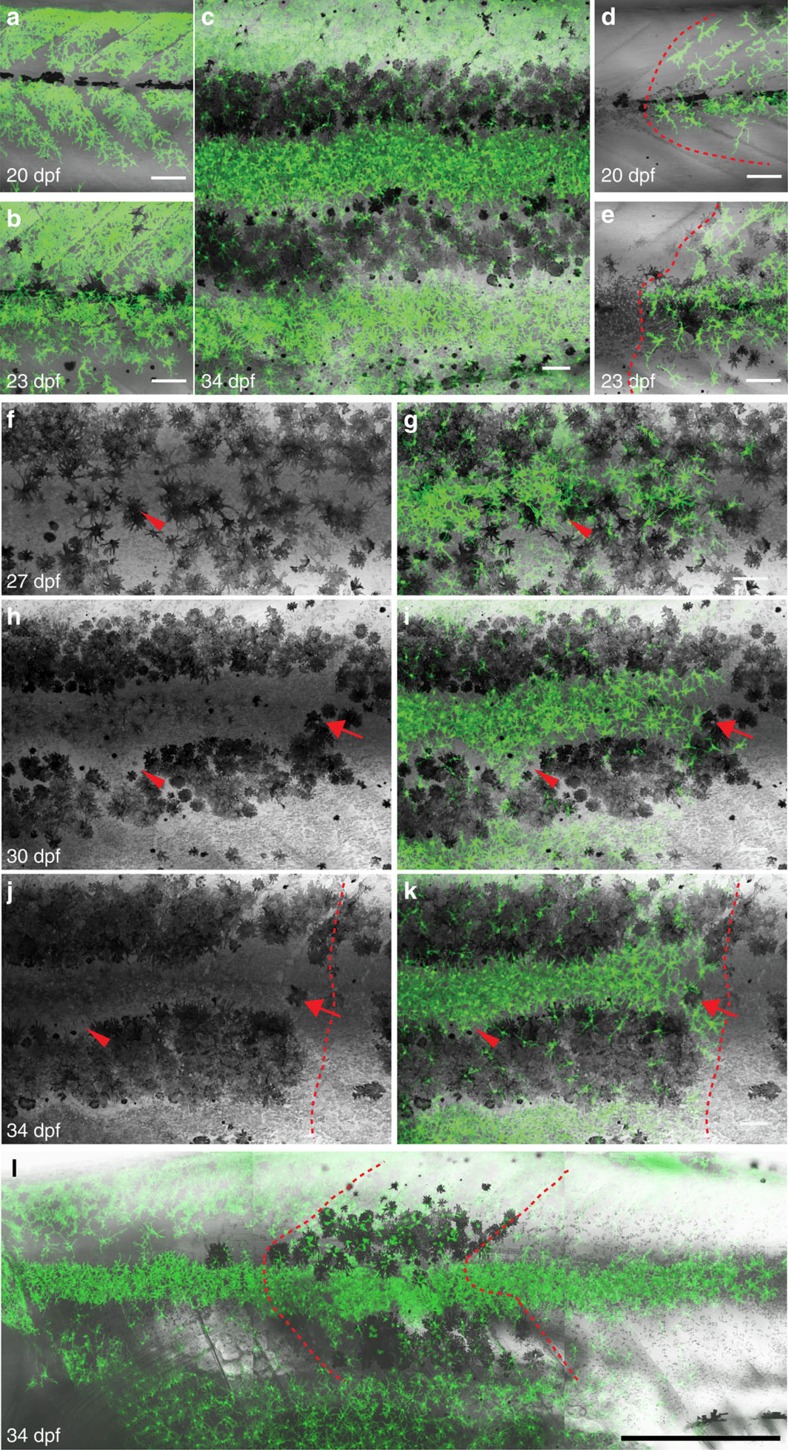
Local rescue of xanthophore numbers and organization by xanthophores in *pfeffer* hosts. (**a**–**c**) Restoration of normal number and organization of xanthophores in *pfeffer*. (**d**,**e**) Xanthophores spread into neighbouring xanthophore-devoid segments as coherent net. Dashed red line: border of transplanted cluster. (**f**–**k**) Restoration of the striped pattern by interactions between the xanthophore cluster and the host melanophores. (**f**–**k**) Widely distributed melanophores (black) become organized into stripes on increase in number of xanthophores and their compaction in the light stripe region. Red arrow: a cluster of melanophores that is cleared over time; red arrowhead: xanthophores retracting from dark stripe region. Dashed red line: border of the cluster showing xanthophores-positive area with rescued pattern on the left, no rescue on the right. (**l**) Chimera obtained by transplantation of *Tg*(*pax7:GFP*) *into pfeffer*;*brass*. The xanthophore cluster covers 17 metamers, whereas the melanophores cluster covers 4 metamers. Xanthophores—green, *Tg*(*pax7:GFP*). Dashed red line: border of melanophores cluster. Scale bars, **a**–**k**: 100 μm, **l**: 250 μm.

Closer analysis revealed that the donor-derived xanthophores divided rapidly in the *pfeffer* hosts at a rate of more than two cell divisions per 10 days, and spread in all directions, most commonly along the myosepta both horizontally and vertically (Fig. 2a–c,d,e). The xanthophores at the border of the clusters collectively migrated into neighbouring regions devoid of xanthophores, thus increasing their coverage (Fig. 2d,e). In several cases individual clusters fused and covered the fish completely. When donor xanthophores encountered host iridophores in the light stripe, they changed in shape to become compact (as in wild type) (Fig. 2c). In *pfeffer* mutants, melanophores form small spots; however, when they encountered donor xanthophores, the melanophores increased in number and locally restored the striped pattern through local migration out of the light stripe region and accumulation in the dark stripe region (Fig. 2f–k). Ectopic melanophores in the mutant areas devoid of xanthophores disappeared on arrival and compaction of donor-derived xanthophores (Fig. 2f–k).

**Figure 3:**
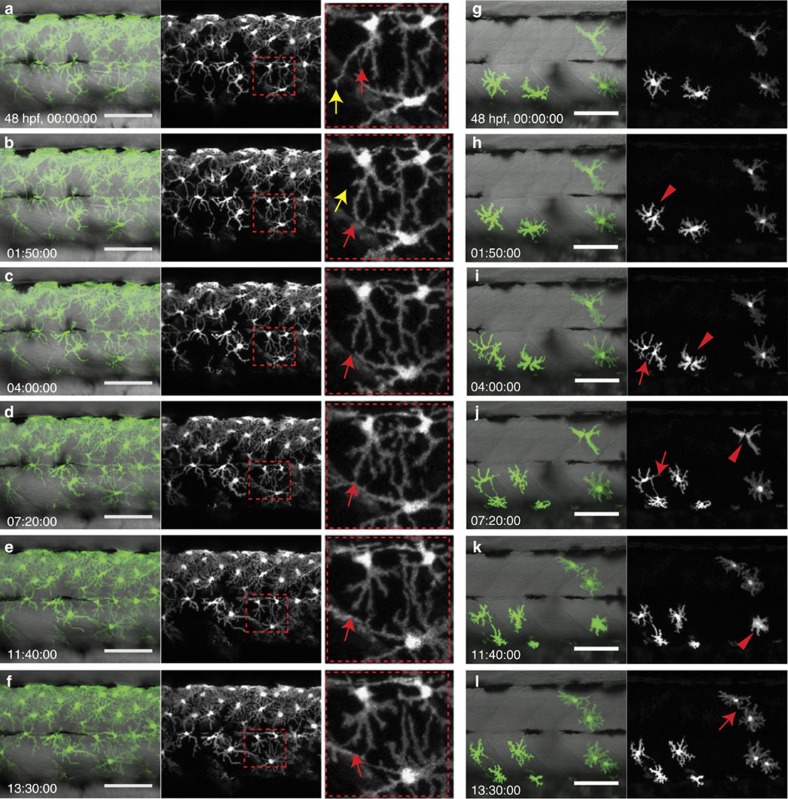
Interaction of xanthophores in larval stage. (**a**–**f**) Live-imaging of wild-type xanthophores—green; *Tg*(*pax7:GFP*), shows that cell numbers remain constant during larval development. (Same images in grey scale). Magnification of red dotted boxes: dynamic cell–cell contacts occur by extension (red arrows) and retraction (yellow arrows) of filopodia. (**g**–**l**) Live-imaging of xanthophores—green; *Tg*(*pax7:GFP*) in *pfeffer* chimera. Transplanted xanthophores divide, for better visibility same image in grey scale (red arrowheads) and extent filopodia to each other (red arrow). Scale bars, **a**–**l**: 100 μm.

Our observations show that donor-derived xanthophores, in the absence of residual xanthophores in *pfeffer* hosts, begin to proliferate during embryonic stages. We confirmed this through live-imaging of xanthophore behaviour in both wild type and *pfeffer* chimeras during late embryogenesis at 48–60 hpf; (Fig. 3; Supplementary Movies 1, 2, 3). Xanthophores in wild-type environments extended dynamic filopodia that allowed them to contact and continually probe each other (Fig. 3a–f: filopodial retraction—yellow arrow, filopodial extension—red arrow; Supplementary Movie 1). Similarly, donor-derived xanthophores rarely changed their location or underwent cell division when transplantated into wild-type embryos (Supplementary Movie 2). In contrast, donor xanthophores in *pfeffer* hosts underwent cell divisions (arrowheads in Fig. 3g–l) and extended dynamic filopodia (arrows in Fig. 3g–l) to explore their environment, and they moved into regions devoid of xanthophores (Fig. 3g–l; Supplementary Movie 3).

To test if the absence of xanthophores in *pfeffer* can also influence melanophore spreading, we transplanted wild-type cells labelled with *Tg*(*pax7:GFP*) into *pfeffer*;*brass* mutants, which lack xanthophores and have unpigmented melanophores^26^,^27^. This allows a clear distinction between donor- and host-derived xanthophores and melanophores. Melanophore clusters did not spread farther than 4 metameres (see below), whereas the xanthophores spread along the anterior–posterior (AP) axis as described above (Fig. 2l). This suggests that the spreading is cell-specific to xanthophores rather than a general property of the *pfeffer* mutation.

These results indicate that the proliferation rate of xanthophores is regulated by competitive homotypic interactions. The xanthophores expand in areas devoid of xanthophores by cell proliferation and by short-scale cell movement. In hosts lacking xanthophores the donor-derived clusters expand in all directions as a coherent net, maintaining normal distances and close contact between cells, rather than dispersing into the space devoid of xanthophores. This indicates mutual interaction and attraction between xanthophores.

### Homotypic interactions among melanophores

**Figure 4:**
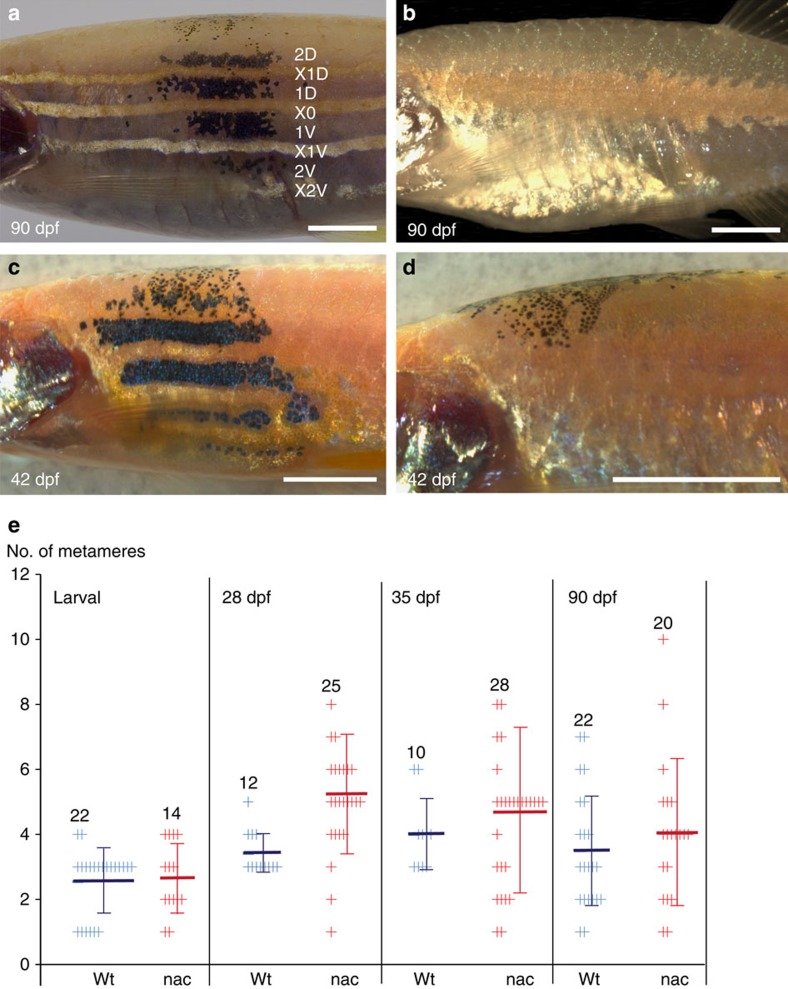
Transplanted melanophore clusters in control (*albino*) and *nacre* chimeras. The organization of wild-type melanophores (black) in (**a**) *albino* and (**c**,**d**) *nacre* hosts. Adult *nacre* fish lacking melanophores (**b**). Scale bars, **a**–**d**: 250 μm. (**e**) Quantification of the number of metamers occupied by wild-type melanophores in the body stripes in *albino* control (blue) and *nacre* (red) hosts. The number of clusters analysed is depicted in the graph. Student's *t*-test (Welsh corrected) revealed significant differences between the size of wild type and *nacre* melanophore clusters at 28 dpf: (wild type, *n*=12 clusters, 7 fishes; *nacre*, *n*=25 clusters, 17 fishes, *P*≤0.0002), at larval stages: (wild type, *n*=22 clusters, 10 fishes; *nacre*, *n*=14 clusters, 20 fishes, *P*=0.7892), 35 dpf: (wild type, *n*=10 clusters, 6 fishes; *nacre*, *n*=28 clusters, 17 fishes, *P*=0.2512 and adult (>90 dpf): (wild type, *n*=22 clusters, 9 fishes; *nacre*, *n*=20 clusters, 17 fishes, *P*=0.3840) no significant difference could be shown. Horizontal lines in dark blue (wild type) and red (*nacre*) indicate the mean value and the error bars represent standard deviation. Student's *t*-test (Welsh corrected) was used to determine the *P*-value.

We analysed the melanophores in normal development using the dark melanin pigmentation, lacking in *albino* recipient fish, as a marker (Fig. 4a). Transplantation of blastomeres labelled with *Tg*(*βact:GFP*) into *albino* hosts yielded 22 melanophore clusters in nine fishes, which were followed into adulthood. Along the AP axis the clusters spread 2–4 segments on average. At the margins of the cluster, the donor-derived black melanophores intermingled with the unpigmented melanophores of the host. Clusters of donor-derived melanophores contributed to several dark stripes along the dorsoventral body axis as described before^6^ (Supplementary Fig. 2). Melanophore clusters in a number of cases (7/28) contributed to all four stripes including the scales, and dorsal and anal fins, whereas 16 clusters contributed to between 1–3 stripes at the age of 3 months (Supplementary Fig. 4). Four clusters of melanophores appeared exclusively in the caudal fins of the fish. These observations are consistent with the known behaviour of melanophores and their origin in a wild-type context.

**Figure 5:**
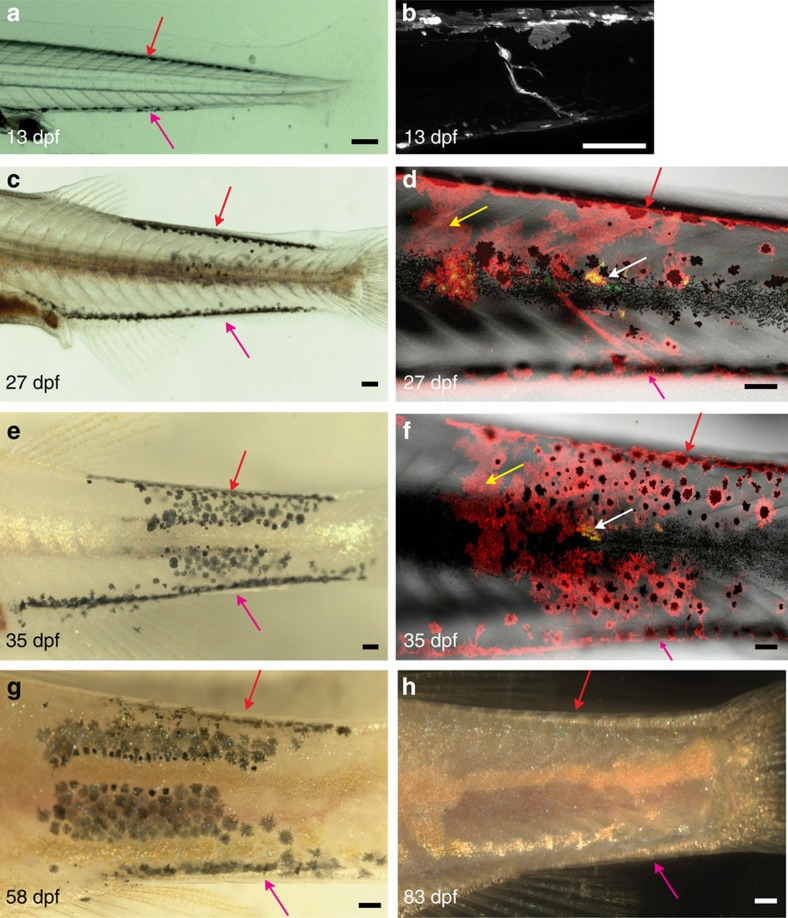
Behaviour of melanophore clusters in *nacre* host. Clusters of wild-type melanophores (*Tg*(*TDL358:GFP*; *sox10:mRFP*)) in *nacre* followed through metamorphosis by (**a**,**c**,**e**,**g**,**h**) bright-field microscopy and (**b**,**d**,**f**) confocal imaging. (**a**–**h**): Dorsal and ventral streaks of melanophores are visible (red and pink arrows). These melanophores gradually disappear. (**e**–**g**) On 13 and 27 dpf confocal microscopy shows a DRG labelled within the melanophore cluster. (**b**,**d**) *Sox10:mRFP* labels clusters of xanthophores (yellow arrows), (**d**,**f**) *TDL358:GFP* labels iridophores (white arrows). (**e**–**g**) The melanophore cluster extends over about six segments. (**h**) At 83 dpf these melanophores disappear completely leaving a ghost dark stripe pattern surrounded by light stripes. Green plus red: iridophores, red only: xanthophores, red plus black: melanophores. Scale bars, **a**–**h**: 100 μm.

Blastomere transplantation into *nacre*^13^ allowed us to track melanophores in the absence of endogenous melanophores (Fig. 4b). A total of 57 clusters of black, donor-derived melanophores were followed in 20 *nacre* fishes up to 3–4 months. As observed in the control (*albino*) chimeras, the donor-derived melanophores contributed to several dark stripes along the dorsoventral body axis (Fig. 4c; Supplementary Fig. 3). In 6 cases out of 57, we found melanophore clones contributing to all four dark stripes as well as pigmentation in scales and anal and dorsal fins. The clusters on the body, although larger than in wild-type chimeras (*albino*; Fig. 4a), remained restricted along the AP body axis, spanning 6 metameres on average (Fig. 4c–e), which is not statistically different from wild-type (*albino*) transplants. As observed previously^12^,^17^,^18^, in *nacre*, the stripes were completely restored in chimeric areas, with a dense sheet of donor-derived melanophores, strictly separated from light stripe regions (Fig. 4c). Intriguingly, clusters started shrinking after 35 dpf and by 4 months, 64% of all clusters in *albino*, and 33% of the clusters in *nacre* disappeared (Fig. 5, cluster in *nacre*). The reason for the late death of donor melanophores in chimeras is unknown. It suggests that in the chimeras the stem cells providing a regular replacement of melanophores have not been maintained.

In *nacre* transplants, a series of larval melanophores on the ventral (30% of all clusters) and the dorsal side (24% of all clusters) of the fish were frequently observed (Fig. 5a–g). These clusters spanned on average 12 metameres and were present from early larval stages through 35 dpf when they started to disappear (Fig. 4d). Figure 5 shows an example of the development of melanophore clusters, labelled with *Tg*(*TDL358:GFP*; *sox10:mRFP*) in *nacre*. *Tg*(*TDL358:GFP*) labels iridophores, whereas *Tg*(*sox10:RFP*) labels all neural crest-derived tissue. On 13 dpf, streaks of larval melanophores were present both dorsally and ventrally (Fig. 5a), and a confocal scan on the same time point shows the presence of a *sox10:mRFP*-labelled DRG (Fig. 5b). At 27 dpf adult red fluorescent protein (RFP)-labelled melanophores appeared flanking the horizontal myoseptum (Fig. 5c,d), along with donor-derived xanthophore and iridophore clusters (Fig. 5d). At 35 dpf the first dark stripes 1 dorsal (D) and 1 ventral (V) were formed (Fig. 5e). In contrast to the melanophore cluster that spreads over 6 metameres, the xanthophore and iridophores clusters remained restricted to 1–2 metameres (Fig. 5f). On 58 dpf the larval melanophore streaks on the dorsal and the ventral side started to disappear, whereas the melanophores in stripes 1D and 1V persisted (Fig. 5g). At 83 dpf all melanophores have disappeared leaving empty areas between intact light stripes (Fig. 5h).

In summary, we observe that host melanophores have little influence on the dispersal of donor-derived melanophores, and that, in contrast to xanthophores, melanophore clusters in the adult pattern rarely spread laterally over more than six segments. Instead, donor-derived melanophores remain together, and form prominent stripes of normal melanophore density despite being surrounded by regions devoid of melanophores. This indicates that in the absence of residual melanophores, an increased number of melanophores reach the skin, and that melanophores attract each other.

### Homotypic interactions among iridophores

**Figure 6:**
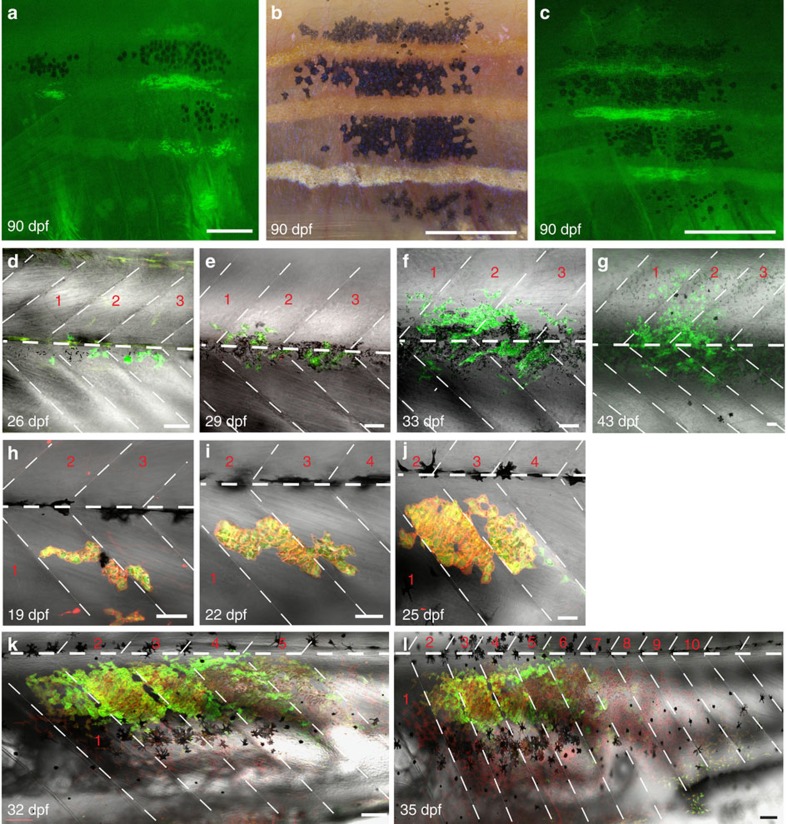
Behaviour of iridophores in control (*albino*) and iridophore-deficient (*shady*) hosts. (**a**–**c**) Iridophore and melanophores clusters (derived from *Tg*(*ß-act:GFP*)) in control (*albin*o) analysed with fluorescence (**a**,**c**) and bright-field microscopy (**b**): at 90 dpf iridophores and melanophores cover several stripes spanning between 1 and 4 metameres. (**d**–**g**) Confocal images of (*Tg*(*TDL358:GFP*))-labelled iridophore clusters in control (*albino*): iridophores appear in three adjacent metameres at 26 dpf and multiply within the same metameres. (**h**–**l**) (*Tg*(*TDL358:GFP*; *sox10:mRFP*)) cluster in *shady* covers 10 metameres at 35 dpf. Dashed white lines indicate vertical and horizontal myosepta. Red numbers indicate metameres. Scale bars, **a**–**c**: 250 μm, **d**–**l**: 100 μm.

The labelling of donor cells with *Tg*(*βact:GFP*) allowed the simultaneous detection of iridophore- and melanophore clusters in chimeric animals in *albino* hosts (Fig. 6a–c). Iridophore clusters displayed a dorsoventral orientation contributing to several light stripes, much like the Cre-induced Sox10 clones previously analysed^5^. In these chimeras progenitors of melanophores as well as iridophores have been transplanted. Transplanting *Tg*(*TDL358:GFP*; *sox10:mRFP*) into wild-type hosts revealed that iridophore clusters begin divisions after the onset of metamorphosis (19–21 dpf). They formed the first light stripe together with the xanthophores (Fig. 6d–g). We followed six clusters through metamorphosis until 40 dpf, showing that the clusters spread over 1–4 metamers and the cell number of the clusters increased from 1–2 cells up to 300 cells within 17 days, which represents a doubling of cell numbers every 3–4 days.

Due to low survival rates of *shady* chimeras, only few clusters of iridophores developing in the *shady* mutant^16^, which lack adult iridophores, could be followed over longer time periods. In the absence of host iridophores, donor-derived iridophores were able to spread laterally, in one case the cluster spanned 10 metameres at 35 dpf, which was never observed in wild type (Fig. 6h-l). This clone showed around 600 mature iridophore cells, whereas iridophore clones in wild type had only around 100 cells at the same time point.

### Regeneration of chromatophores after stem cell depletion

Analysis of homotypic interactions among iridophores was hampered by a low survival rate of *shady* chimaeras. Other iridophore mutants, such as *rose* and *transparent*, have residual iridophores and hence are not suitable for our analysis. To obtain more information on the autonomous behaviour of iridophores and melanophores, we disrupted the formation of melanophore and iridophore stem cells by inhibiting the erb-b2 receptor tyrosine kinase (ErbB) pathway. ErbB signalling is required to establish the DRGs during the migration of neural crest cells in the early embryo^6^,^28^. To reduce ErbB3 activity in a temporally controlled manner, we used the small molecule inhibitor PD168393 at a concentration that results in the absence of several of the DRG-located stem cells of melanophores and iridophores causing gaps in the striped pattern^6^,^28^. This allowed us to study the behaviour of surviving iridophores and melanophores during the regeneration of the striped pattern in the gaps.

**Figure 7:**
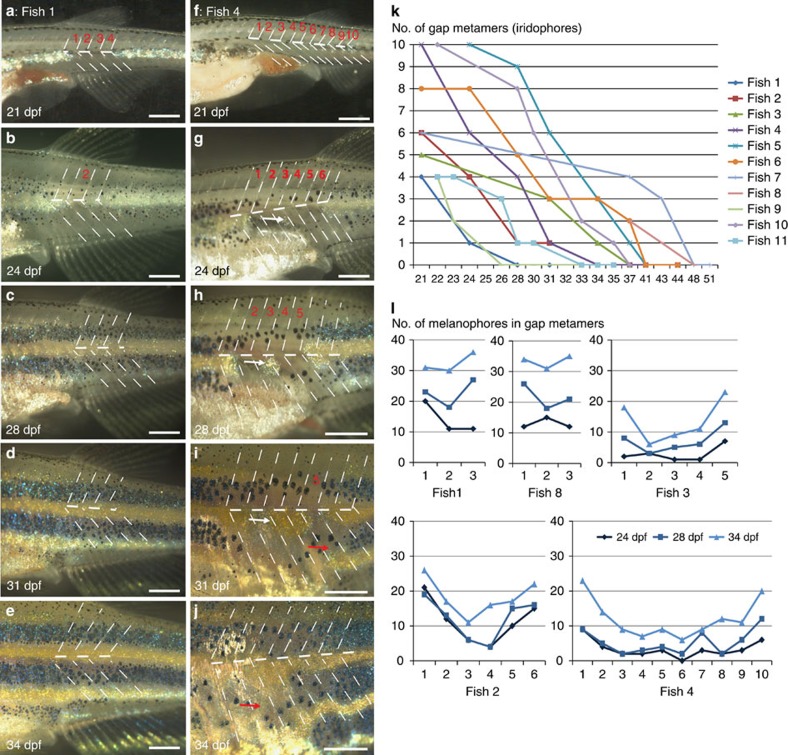
Regeneration of iridophores and melanophores after ErbB inhibition. Treatment with PD168393 during embryogenesis (see methods) results in (**a**–**j**) gaps of different sizes (1–12 metamers) in the striped pattern of metamorphosing fish. Overall 15 fishes carrying gaps between 3 and 12 metamers were imaged through metamorphosis. Red numbers (**a**–**i**) indicate the segments that are devoid of iridophores at the onset of bright-field imaging. Dashed lines indicate vertical and horizontal myosepta. On 24 dpf (**g**) a spot of regenerating iridophores appears in the middle of the gap and it expands laterally (white arrow in **g**,**h**,**i**). (**k**) Time (in days) taken to fill the gaps of variable sizes in the stripe pattern. (**l**) Increase in numbers of melanophores in individual fish (*n*=5) during regeneration of gap metamers at three different time points, 24, 28 and 34  dpf. Scale bars, **a**–**j**: 250 μm.

In many of the drug-treated fish, gaps in the striped pattern were observed at 21 dpf: they displayed regions with a reduced number of larval melanophores, and absence of dense iridophores forming the first light stripe at the horizontal myoseptum extending between 3 and 12 metameres (Fig. 7a,f; Supplementary Fig. 5). Fish with gaps in the stripes were followed from 21 dpf every 3–4 days until 34 dpf (Fig. 7). We observed that 4–6 segment large gaps were completely filled by iridophores coming from adjacent segments (Fig. 7a–e) and the pattern regenerated within 7–10 days (quantification in Fig. 7k). Large gaps (∼10 metameres on 21 dpf) were also filled in by spreading iridophores, but the recovery took longer (Fig. 7f–k). The striped pattern was not repaired perfectly resulting in wavy light and dark stripes on 34 dpf. In some cases (*n*=7) we observed that iridophore clusters appeared in the middle of the gaps (white arrows in Fig. 7g–i) spreading in all directions and resulting in a broader light stripe. In smaller gaps the cell number of melanophores in metameres recovered fully within 10 days yielding between 30 and 35 melanophores per metamere, the same number as compared to the control (the striped regions outside the gap) (Fig. 7l fish 1, 8; Supplementary Table 1), whereas in the bigger gaps on 34 dpf the melanophore number was still lower (<10 melanophores per metamere Fig. 7l, fish 2, 3 and 4; Supplementary Table 1). In these larger gaps, only the segments adjacent to the striped region recovered significantly.

**Figure 8:**
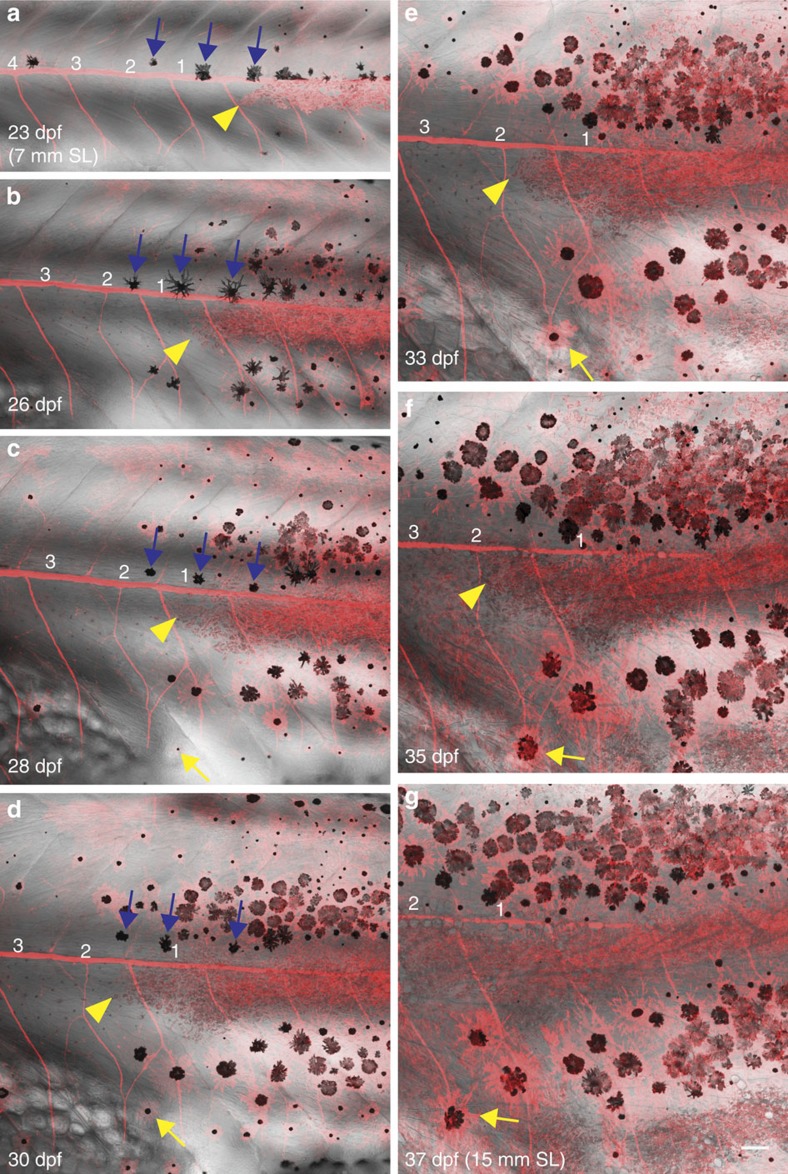
Behaviour of iridophores and melanophores during pattern regeneration after ErbB inhibition. (**a**–**g**) Confocal images of *Tg*(*sox10:mRFP*) animal developing with a gap (indicated by number 1–4) in the stripe pattern. Existing iridophores (yellow arrowheads) extend laterally into the prospective light stripe region that is devoid of iridophores and fill the region. New melanophores appear near the margins of the gap and locally restore the pattern. Occasionally, melanophores display short-scale movement (yellow arrows). Blue arrows: melanophores situated along the horizontal myoseptum that move dorsally to join dark stripes on regeneration of the light stripe by iridophores. Iridophores—red (*Tg*(*sox10:mRFP*). SL, standard length of fish. Scale bars,100 μm.

**Figure 9:**
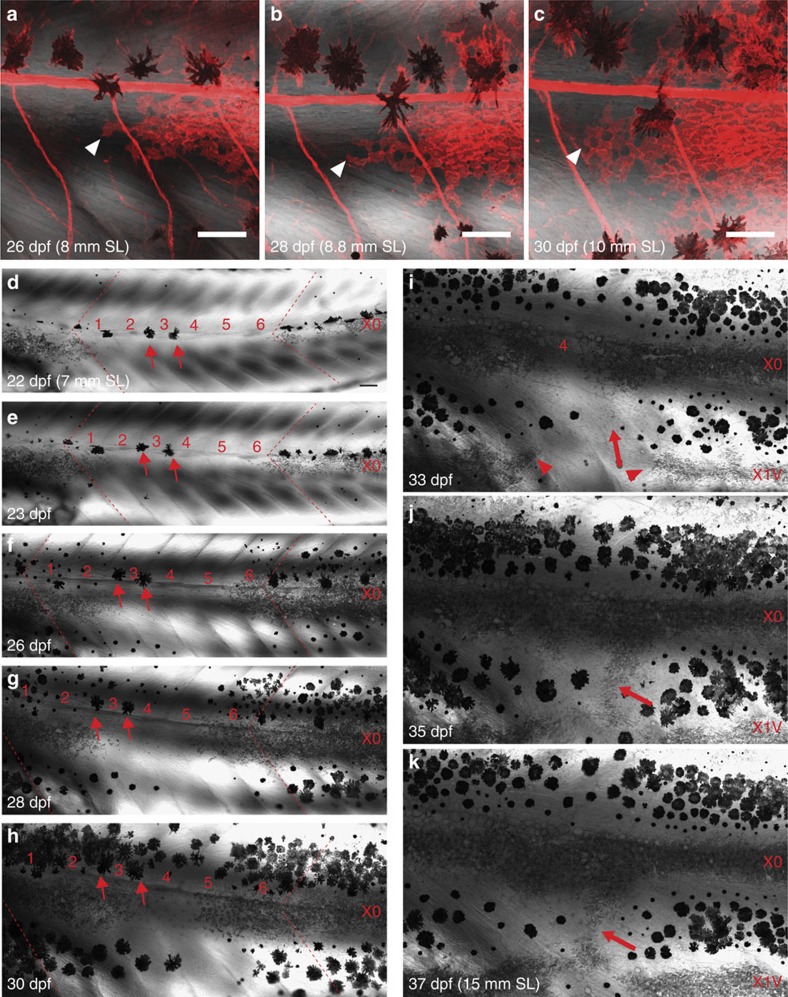
Iridophore disperse laterally to occupy iridophore-devoid regions. (**a**–**c**) Iridophores (white arrowheads, *Tg*(*sox10:mRFP*)) extend to the empty segment on the left and increase in number. (**d**–**k**) Gap closure in an animal lacking iridophores in six segments at 22 dpf (numbered 1–6). Red dashed lines indicate the original borders of the gap. Small arrows in **d**–**h**: larval melanophores remain stable within the gap. (**e**) Iridophores that are present outside the gap begin to invade the iridophore-devoid segments and within a day, iridophores can be seen in segment 1 and 6. (**f**–**h**) By lateral movement, iridophores recover in segments 1–3 and 5–6, only the segment 4 remains devoid of iridophores. (**i**) The first light stripe, X0 lacks iridophores in the segment 4 and remains interrupted. Corresponding region in the newly formed light stripe also lacks iridophores (red arrowheads). The dark stripe region does not develop sufficient melanophores and subsequently iridophores begin to migrate vertically (big red arrow) invading the dark stripe region. (**j**,**k**) Vertically migrating iridophores lead to interruption of the dark stripe region by the light stripe region. SL, standard length of fish. Scale bars,100 μm.

To visualize the behaviour of surviving iridophores and melanophores during pattern regeneration, we performed drug-mediated ErbB inhibition on animals carrying *Tg*(*sox10:mRFP*) and imaged metamorphic fish (Figs 8, 9; Supplementary Fig. 5). Iridophores at the margins of the gaps migrate laterally, occupying iridophore-devoid regions of the prospective first light stripe (arrowheads in Fig. 8). Thus by proliferation and lateral dispersal, iridophores closed the gaps of 4–6 metamers completely within 10–12 days (graph in Fig. 7k). Iridophores at the margins of the gaps displayed the morphology of loose iridophores, and proliferated and migrated (Fig. 9a–c). Iridophores that are behind the migratory iridophores became densely packed and acquired the morphology of light stripe iridophores. In animals developing with large gaps (∼10 segments), occasionally regenerating iridophores could be seen in the middle of the gaps (Fig. 7g-i; Supplementary Fig. 5). These iridophore clusters were similar in size to iridophore clones originating from stem cells^5^, suggesting a regeneration potential for the progenitors.

Melanophores also regenerated from the margins of the gaps. Although a few melanophores displayed lateral movement (yellow arrow in Fig. 8c–g), most melanophores appeared *de novo*. This suggests that the new melanophores originate from unpigmented melanoblasts, which are regenerated by stem cells in the segments at the margins of the gaps. The melanophores exhibited limits in movement; in Fig. 8, three melanophores situated along the horizontal myoseptum (blue arrows in Fig. 8a–d), displayed short-scale movement towards the dorsal stripe region on the arrival of iridophores in the adjacent light stripe region. The ErbB inhibition did not affect xanthophores (Supplementary Fig. 5).

A closer analysis of developing animals that had a gap in the first light stripe, allowed us to study the formation of an interrupted stripe pattern (Fig. 9d–k). In an animal with an initial gap of six segments, five segments were filled by lateral migration of iridophores within 10 days (Fig. 9d–i). Interestingly, a break in the first light stripe X0 corresponded to a break in newly forming light stripe X1V (red arrowheads in Fig. 9i). This is consistent with a clonally-related origin of iridophores along the dorsoventral axis^5^. Some larval melanophores persisted in the gaps (red arrows in Fig. 9d–h), however, the melanophore numbers did not recover as fast as iridophores, leading to a melanophore-deprived region in the dark stripe (Fig. 9d,e). Subsequently, this melanophore-deprived region was invaded by iridophores from the first (X0) and the newly forming (X1V) light stripes, leading to interruptions of the dark stripe by light stripe regions (Fig. 9i-k). We conclude that the lateral spreading of the melanophores, in contrast to that of the iridophores is constrained.

### Regeneration of xanthophores after cell-specific ablation

**Figure 10:**
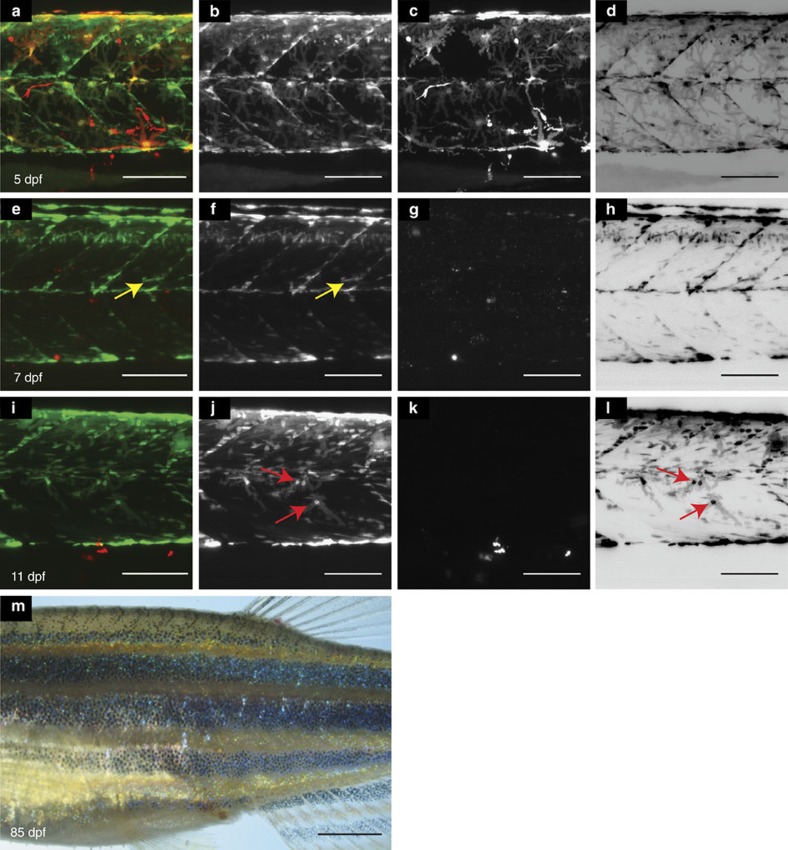
Xanthophore regeneration following drug-mediated ablation. (**a**–**d**): Larva at 5 dpf, xanthophores: green (*Tg*(*pax7:GFP*) and red: *Tg*(*fms:Gal4*, *UAS:nfsB-mcherry*). (**e**–**h**): The same region after ablation at 7 dpf: yellow arrow: (*Tg*(*pax7:GFP*) labels muscle tissue and their precursors^24^. (**i**–**l**): same region at 11 dpf. Red arrow: regenerated xanthophore. (**a**,**e**,**i**): merge; (**b**,**f**,**j**): (*Tg*(*pax7:GFP*) in grey scale; (**c**,**g**,**k**): *Tg*(*fms:Gal4*, *UAS:nfsB-mcherry*) in grey scale; (**d**,**h**,**l**): (*Tg*(*pax7:GFP*) in inverted grey scale. (**m**): Adult fish after ablation displaying a normal pattern. Scale bars, **a**–**l**: 100 μm, **m**: 250 μm.

To test the regenerative potential of xanthophores, we ablated larval xanthophores at 5 dpf by nitroreductase-mediated cell ablation^29^ in *Tg*(*fms:gal4*; *UAS:nfsB-mCherry*) larvae. To efficiently trace the xanthophores, they were carrying *Tg*(*pax7:GFP*) (which also labels muscle stem cells). Following metronidazole treatment for 24 h, nitroreductase expressing xanthophores were efficiently ablated without affecting the surrounding architecture (Fig. 10 a–l). To visualize the extent of xanthophore regeneration, we tracked the ablated regions using confocal microscopy. By 10 dpf regenerated green fluorescent protein (GFP)-labelled xanthophores could be observed in each segment. However, unexpectedly, regenerated xanthophores did not show the *fms:gal4*-mediated *nfsB-mCherry* expression, suggesting silencing of the transgene. When grown to adulthood, fish displayed a normal formation of the striped pattern indicating that xanthophores were fully restored (Fig. 10m). We suggest that the regeneration occurs from xanthophores originating from multipotent iridophore/melanophore stem cells^5^.

## Discussion

The formation of the striped colour pattern in zebrafish involves several cell behaviours, including pigment-cell proliferation, dispersal and spatially controlled cell-shape transitions in the skin^1^,^5^,^8^,^23^. In this study, we find a role for homotypic competition in regulating pigment-cell proliferation, dispersal and tiling in the skin for all three chromatophore types. In the absence of competing cells within a layer in the skin, donor-derived clusters of xanthophores and iridophores proliferate at a faster rate than in their normal surrounding and cover larger regions of the skin. Mutant analysis indicates that specification, proliferation and maintenance of pigment cells depend on individual receptor–ligand systems; Kit signalling for melanophores, Ednr and Ltk signalling for iridophores, and Csf1r signalling for xanthophores^12^,^14^,^15^,^16^,^30^,^31^,^32^. Whereas the pigment cells express the respective receptor, the ligands are presumably functioning as trophic factors produced by surrounding tissue. Competition for the ligands may in part be responsible for the regulation of pigment-cell proliferation in normal development.

Competitive interactions among pigment cells result in an overall directionality in the pigment-cell migration that determines the dorsoventrally orientated clonal expansion of the pigment cells. During normal development, in the presence of pigment cells of the same type, clusters of all three pigment-cell types spread in dorsoventral direction as the fish grows ((ref. 5); Fig. 6a–c; Supplementary Figs 1, 2). The clusters contributed to most if not all stripes in a narrow patch along the dorsoventral axis. Spreading along the anteroposterior direction was less pronounced, and the clusters contributed to between 1 and 4 adjacent segments. This reveals that in normal development, the pattern is stitched together by adjacent clones derived from stem cells at the DRG of neighbouring metamers (iridophores and melanophores), and from larval xanthophores. The lateral extension of the clones is restricted by homotypic competition. Thus, the dorsoventral orientation of the pigment-cell clones observed in normal development is the result of competitive interactions between cells from neighbouring segments and not the product of a hypothetical, morphogen-based directed migration.

As reported earlier^12^,^18^, the introduction of the missing cell type into mutants lacking a given pigment-cell type locally restores a normal pattern. The incoming cells in the spreading clusters induce cell-shape changes in the adjacent cell layers such that a normal stripe pattern is formed while the clusters grow. This involves extensive shape changes in the pigment cells of the host as well as the donor. Our high-resolution images confirm that there is a constant adjustment with mutual interactions between the three cell types during stripe formation. In conclusion, heterotypic interactions between the three layers regulate cell-shape transition, whereas the homotypic interactions we observe regulate pigment-cell dispersal in a coherent two-dimensional sheet within one layer.

Strikingly, in adult chimeras, the patches of donor-derived pigment cells are coherent and have normal densities. This indicates that there is an intrinsic tendency for pigment cells to remain together observing normal distances, rather than dispersing into regions devoid of their cell type. We suggest that the cell–cell interactions are mediated by direct cellular contacts. Live-imaging of the behaviour of xanthophores in larvae (Fig. 3) shows that xanthophores maintain a dynamic contact with one another, and proliferate in the absence of neighbouring xanthophores. The xanthophore filopodia are highly dynamic actin-rich protrusions^33^, which may allow xanthophores to probe their environment and contact neighbouring xanthophores, thus maintaining a sheet-like xanthophore cover in the skin through cell division and migration into xanthophore-devoid regions. In the growing skin, increasing the distances between neighbouring xanthophores may trigger cell divisions such that a regular spacing is maintained.

Iridophores spreading in *shady* chimeras, or laterally migrating into gaps devoid of iridophores also acquire a loose migratory shape but stay in direct contact with one another rather than dispersing. This behaviour is also observed in normal development in which iridophores during metamorphosis populate the skin as a loose net of interconnected cells spreading dorsally and ventrally^5^.

The melanophores in stripes formed in *nacre* chimeras also maintain normal distances to one another. In normal development, melanophores extend long cytoplasmic protrusions allowing them to contact each other while filling in the dark stripe region^5^. This suggests that the spacing of melanophores in the stripes is mediated by direct cell contacts between melanophores. Mutant analyses, however, indicate in addition a strong dependence of melanophore spacing on heterotypic interactions, in particular with xanthophores, whereas iridophores and xanthophores display a more autonomous behaviour^12^.

The responses of pigment cells to homotypic interactions, in particular those observed in xanthophores and iridophores, resemble that of neural crest cells, which undergo collective cell migration and display co-attraction despite contact inhibition of locomotion^34^. Contact inhibition of locomotion is a long-established phenomenon observed in cultured cells *in vitro*^35^,^36^,^37^,^38^, and more recently *in vivo*^39^,^40^,^41^. It is suggested that co-attraction and contact inhibition of locomotion act in concert orchestrating collective migration of neural crest cells—contact inhibition of locomotion alone would lead to dispersal of the group into single cells^40^. Co-attraction, in combination with contact inhibition of locomotion allows maintenance of appropriate cell density, local homotypic cell–cell interactions and efficient response to external cues. We hypothesize similar interactions between pigment cells of the same kind during pattern formation—clusters of wild-type xanthophores do not disperse into single cells in absence of xanthophores in the neighbourhood. Instead, these cells maintain appropriate local cell density while the cluster grows by local proliferation and short-scale movement. This is also true for iridophores and melanophores suggesting co-attraction and contact inhibition of locomotion may underlie pigment-cell coverage by homotypic interactions in the skin. Our knowledge of the molecular bases of cell contact-mediated interactions during colour pattern formation is still rudimentary. At the molecular level, interactions between chromatophores have been shown to depend on integral membrane proteins including connexins and potassium channels, suggesting that physical cell contacts between chromatophores are involved and that the directed transport of small molecules or bioelectrical coupling are important for these interactions^21^,^22^,^42^. In *obelix*, encoding the inwardly rectifying potassium channel Kir7.1, melanophore stripes are enlarged, with a lower than normal density of melanophores^18^,^43^. This channel is specifically required in melanophores and may be responsible for their proper spacing. The gap junctions formed by the connexins encoded by the *leopard* and *luchs* genes have been suggested to mediate heterotypic as well as homotypic interactions among melanophores and xanthophores^18^,^22^,^44^. Connexins mediate intercellular communication that may lead to diverse cellular outcomes including oriented cell migration^45^. The Immunoglobulin Superfamily Member 11, mutated in *seurat*, has been reported to mediate aspects of melanophore behaviour including melanophore–melanophore interactions^46^. It is conceivable that connexins and other cell-surface molecules mediate the homotypic competition-dependent proliferation and tiling of the pigment cells. Our experimental paradigm provides an opportunity to assess the molecular regulation of the cellular outcomes of the diverse cell–cell interactions during colour pattern formation.

## Methods

### Zebrafish lines

The following zebrafish lines were used: wild-type (WT, Tübingen strain^47^ from the Tübingen zebrafish stock centre), *albino*^25^, *nacre*^13^, *pfeffer*^18^, *brass*^27^, *shady*^16^,^27^, *Tg*(*fms:Gal4*, *UAS:mcherry*)^48^, *Tg*(*pax7:GFP*)^8^,^24^, *Tg*(*TDL358:GFP*)^49^, *Tg*(*sox10:mRFP*)^5^, *Tg*(*βactin2:GFP*). Zebrafish were raised as described^50^. The staging of metamorphic fish was done as described^12^,^51^. All animal experiments were performed in accordance with the rules of the State of Baden-Württemberg, Germany and approved by the Regierungspräsidium Tübingen.

### Blastomere transplantation

Chimeric animals were generated by transplantation of cells of appropriately labelled blastula stage embryos into wild type and mutant embryos at blastula stage^52^. The number of transplanted cells was monitored using Rhodamine dextran injected in the donor embryos at the one cell stage, and the number of transplanted cells was estimated to be in the range between 1–10 cells. Fishes were raised and analysed at different time points throughout metamorphosis until adulthood.

### Pharmacological inhibition of the ErbB signalling pathway

The ErbB inhibitor-treatment was done as described previously^6^. In brief, 14 somite stage embryos were treated with 10 μM ErbB inhibitor (PD168393) dissolved in E2 for 5 h at 29 °C. The ErbB inhibitor phenotype is similar to that of *erbb3b* (*hypersensitive*/*picasso*) mutants^6^,^28^: during the early stages of metamorphosis the fish develop with regions in the prospective light and dark stripe regions without iridophores and melanophores. Wild-type animals at corresponding stages display contiguous light and dark stripes. We term the melanophore and iridophore-devoid regions as “gaps”. The size of these gaps along the AP axis is variable in both, the *erbb3b* mutants and the ErbB inhibitor-treated animals.

### Genetically-targeted ablation of xanthophores

Targeted ablation of xanthophores was performed as described^29^. In brief, larvae carrying *Tg*(*fms:Gal4*, *Tg*(*UAS:nfsB-mcherry*))^48^ were treated with metronidazole for 24 h at 5 dpf. This results in targeted ablation of xanthophores. *Tg*(*pax7:GFP*)^8^,^24^, a reporter line that labels xanthophores, muscles and muscle progenitors, was used for xanthophore visualization. Xanthophores can be recognized by their characteristic shape and location in the skin. The first imaging of the larvae was done immediately before the drug treatment. Subsequent imaging was done as mentioned in the text.

### Image acquisition and processing

Repeated imaging of zebrafish was performed as described^5^. Images were acquired on Zeiss LSM 780 NLO confocal and Leica M205 FA stereo-microscopes. ImageJ^53^, Adobe Photoshop, Adobe Illustrator and Imaris were used for image processing and analysis. Maximum intensity projections of confocal scans of the fluorescent samples were uniformly adjusted for brightness and contrast. Scans of the bright field were stacked using “stack focuser” plugin and tile scans were stitched in Fiji^54^.

## Additional information

**How to cite this article:** Walderich, B. *et al*. Homotypic cell competition regulates proliferation and tiling of zebrafish pigment cells during colour pattern formation. *Nat. Commun.* 7:11462 doi: 10.1038/ncomms11462 (2016).

## Supplementary Material

Supplementary Information Supplementary Figures 1-5 and Supplementary Table 1

Supplementary Movie 1 Confocal live-imaging of wild-type xanthophores from 48 hpf to 61.5 hpf in transgenic zebrafish (green; Tg(pax7:GFP)). One scan per 10 minutes (81 frames). Xanthophores show dynamic filopodia-mediated cell-cell contact. We were not able to detect any cell division in wild-type situation during this time period (movies no. = 5, cells no. at 48 hpf = 137 and cells no. at 61.5 hpf = 137).

Supplementary Movie 2 Confocal live-imaging of wild-type xanthophores (green; Tg(pax7:GFP) transplanted into wild-type from 48 hpf to 61.5 hpf in transgenic zebrafish. One scan per 10 minutes (81 frames). Transplanted xanthophores show dynamic filopodia. No cell division events were detected in the wild-type situation throughout the duration of the liveimaging (N = 3 movies; number of xanthophores at the start of the movie at 48 hpf = 7; number of xanthophores at the end of the movie at 61.5 hpf = 7).

Supplementary Movie 3 Confocal live-imaging of wild-type xanthophores (green; Tg(pax7:GFP) transplanted into pfeffer from 48 hpf to 61.5 hpf in transgenic zebrafish. One scan per 10 minutes (81 frames). Transplanted xanthophores show long dynamic filopodia in search of cell - cell mediated contact with other xanthophores. We detected cell division of wildtype xanthophores in pfeffer chimeras (N = 4 movies; number of xanthophores at the start of the movie at 48 hpf = 24; number of xanthophores at the end of the movie at 61.5 hpf = 34).
