# Supplementary material for: Homotypic cell competition regulates proliferation and tiling of zebrafish pigment cells during colour pattern formation
Source: Nat Commun. 2016 Apr 27;7:11462. doi: 10.1038/ncomms11462 (PMC4853480; doi:10.1038/ncomms11462)
Supplement: Supplementary Information — Supplementary Figures 1-5 and Supplementary Table 1 [file ncomms11462-s1.pdf]

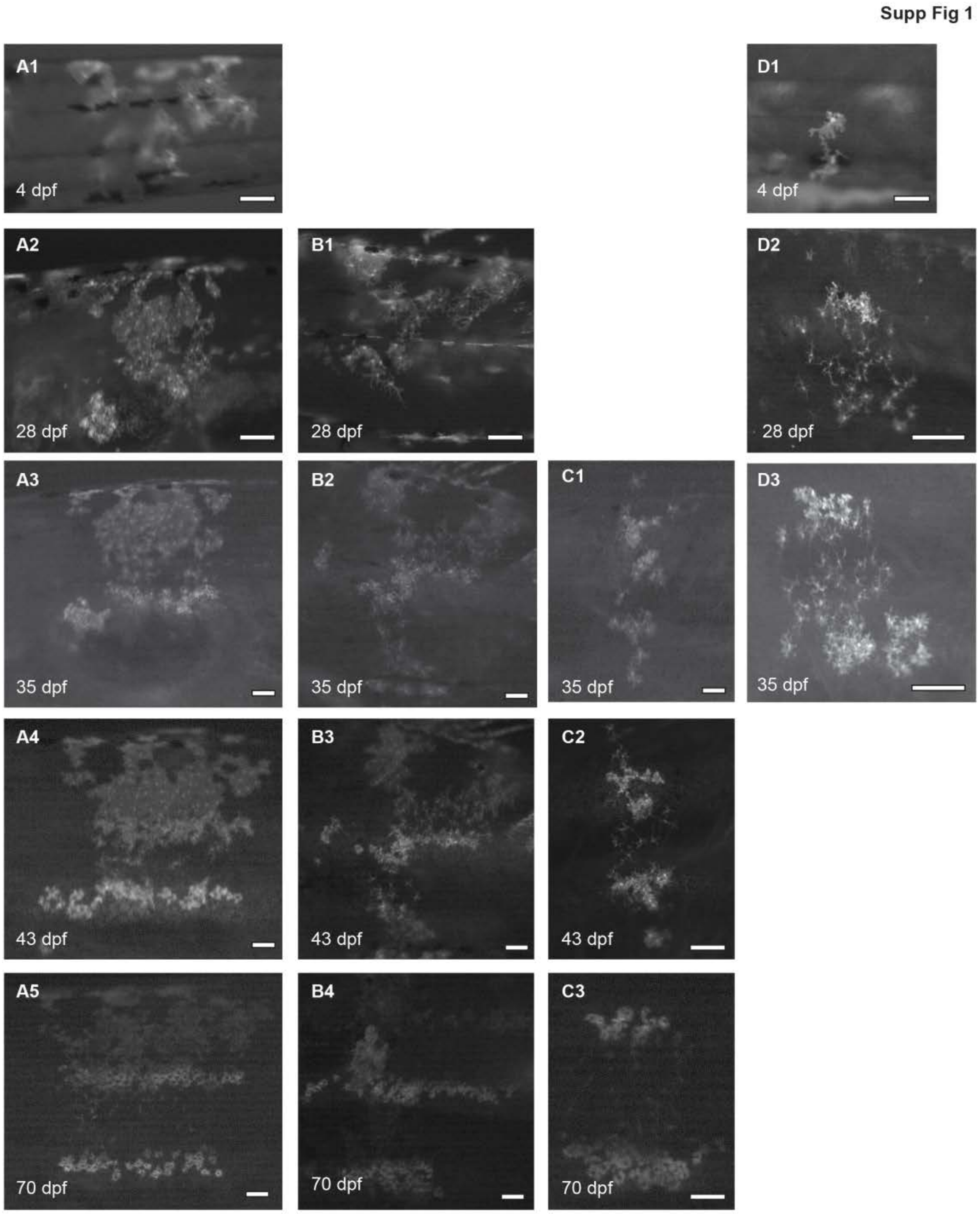

Supplementary figure 1: Examples showing development of xanthophore clusters in wild-type chimeras during metamorphosis. Xanthophore clusters stay restricted between 1 – 4 metamers and contribute to several light and dark stripes along the dorsoventral axis, show small rearrangement until 70 dpf. Shape changes of xanthophores start to happen from the beginning of stripe formation (28 dpf), resulting in stellate and densely-organized cells in the dark and the light stripe regions, respectively. All images are acquired with a Leica M205FA microscope. Scale bars: 100 μm

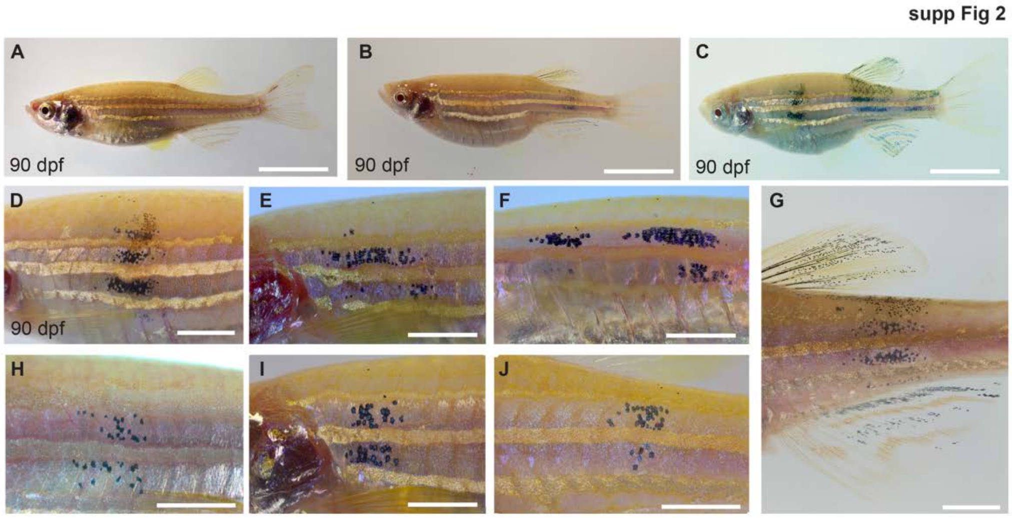

Supplementary figure 2: Examples of melanophores clusters in wild-type chimeras on 90 dpf. (A) *albino* host (B,C) chimeras of wild-type and *albino* hosts. Clusters spread over 1 – 4 metameres and may span the complete body, including the fins along the dorsoventral axis. (D–J) close ups of melanophore clusters in *albino* show that the black donor melanophores are scattered between colourless *albino* melanophores. Additionally melanophores do not always cover all dark stripes. All images are acquired with a Canon 5D Mk II camera. Scale bars A-C: 1 cm; D-J: 250 µm.

supp Fig 3

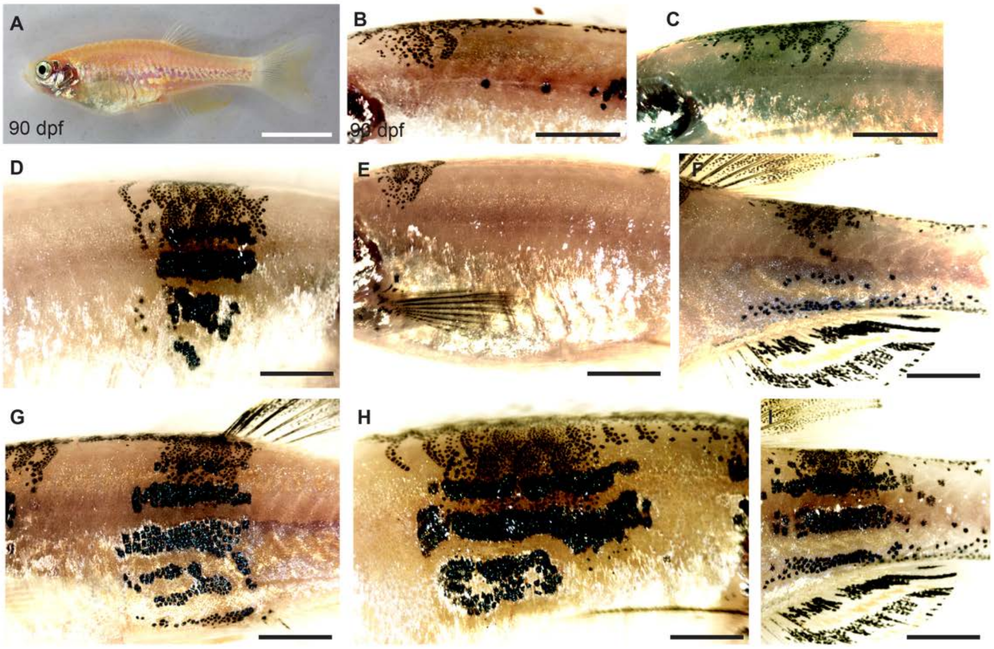

Supplementary figure 3: Examples of melanophores clusters in *nacre* chimeras. (A) *nacre* host (B–I) chimeras of wild-type (*Tg(βAct:gfp)* and *nacre* hosts. (B,C,F;G;I) Melanophore clusters appear at the dorsal side and in the scales (D,E,F,G,H,I) Host melanophores may populate all dark stripes from dorsal to ventral including fins, they may extend over 6 metamers. All pictures were taken with a Leica M205FA microscope and with a Canon 5D Mk II camera.
Scale bars A: 1 cm, B–I: 250 µm.

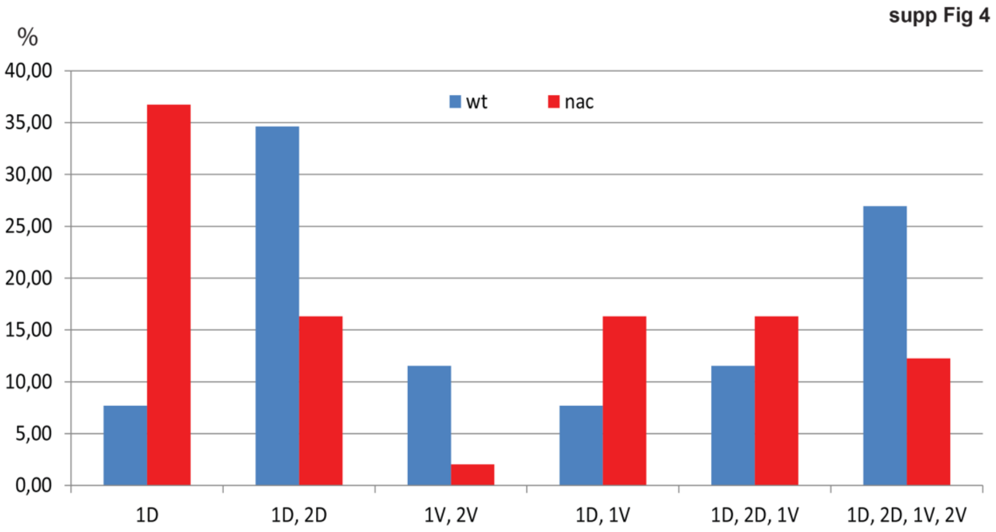

Supplementary figure 4: Striped pattern of transplanted melanophore clusters in wild-type and *nacre* background.

Percentage of wild-type melanophore clusters that contributed to dark stripes along the dorsoventral axis in control (blue) and *nacre* (red) hosts. 28 clusters in control *(albino)(12 fish)* and 58 clusters in *nacre (31 fish)* were analysed at 3 months post fertilisation. Statistical analysis was not done because of low numbers for comparison of the different stripe patterns. Stripe nomenclature as in Frohnhöfer et al., 2013[11].

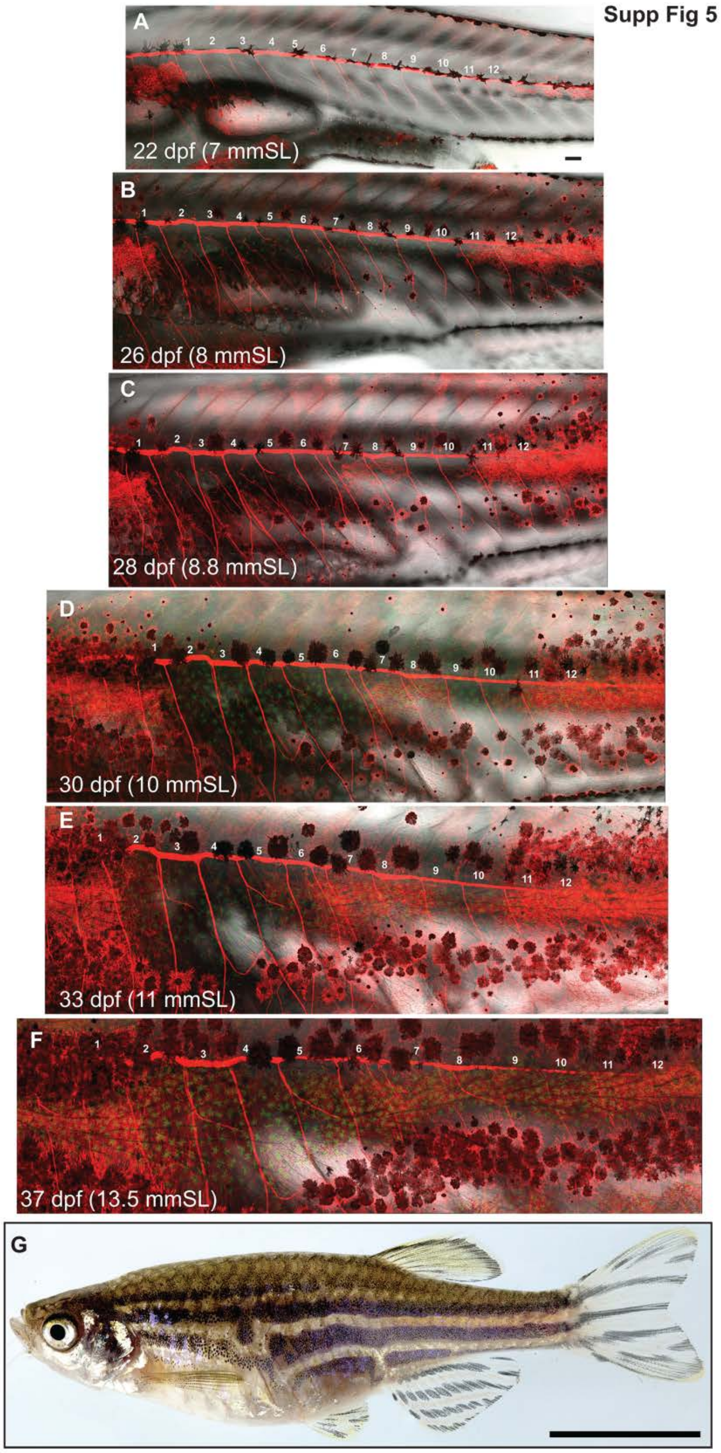

Supplementary figure 5: Regeneration of a large gap stripe pattern caused by ErbB inhibition.

(A-B) An eleven segment-wide gap in the first light stripe is filled initially be lateral movement of iridophores. (C-D) The new melanophores appear but in numbers lower than in the control segments. On 28 dpf in (C), an ectopic iridophore cluster is seen in the middle of the gap in segments numbered 7-8. (D-F) The iridophores from this new cluster begin to spread thus filling

the gap completely on the right side (segments numbered 9-11) where melanophores also catch up in terms of numbers. Stripe pattern is restored in this region. On the left side of the gap (segments numbered 3-5), melanophores fail to appear in numbers comparable to that in the control regions and iridophores of abnormal morphology and organization appear. This region develops wavy stripes of abnormal width. Scale bar in A: 100 µm. (G) Bright field image of the same fish at adult stage, the locally abnormal stripe organization can still be seen (Scale bar=1 cm). Genotype: *Tg(sox10:mRFP)*. Green: xanthophore auto-fluorescence. SL: standard length of fish.

Supplementary Table 1

Melanophore numbers in gap metamers in regenerating fish after ErbB inhibition

		24 dpf	28 dpf	31 dpf	34 dpf
	# gap metameres	# m	# m	# m	# m
Fish 1	1	20	23	25	31
gap: 3 m	2	11	18	24	30
	3	11	27	36	36
Fish 2	1	12	26	22	34
gap: 3 m	2	15	18	27	31
	3	12	21	23	35
Fish 3	1	2		8	18
gap: 5 m	2	3		3	6
	3	1		5	9
	4	1		6	11
	5	7		13	23
Fish 4	1	21	19	21	26
gap: 6 m	2	12	13	17	17
	3	6	6	10	11
	4	4	4	13	16
	5	10	15	18	17
	6	15		24	22
Fish 5	1	9	9	19	23
gap: 10 m	2	4	5	13	14
	3	2	2	12	9
	4	2	3	6	7
	5	3	4	10	9
	6	0	2	6	6
	7	3	8	8	9
	8	2	2	5	12
	9	3	6	10	11
	10	6	12	17	20
Average		7,30	11,57	14,85	18,26
StDev		5,93	8,38	8,34	9,65

Control metameres		24 dpf	28 dpf	31 dpf	34 dpf
		# m	# m	# m	# m
	Average	19,58	19,64	28,04	33,73
	StDev	4,70	3,50	3,43	6,54

Supplementary table 1: Regeneration of melanophores in the gaps caused by ErbB inhibition in comparison with unaffected metameres. Melanophore counts per metamere of 5 treated fish and metamers of untreated controls are shown for 24, 28, 31 and 34 dpf. In comparison with melanophore numbers in control metamers the counts show that in smaller gaps the numbers of melanophores reach their normal number, whereas in larger gaps the number of melanophores of the metamers stays below the expected value.
